# Variances in the Expression Profile of Circadian Clock-Related Genes in Astrocytic Brain Tumors

**DOI:** 10.3390/cancers16132335

**Published:** 2024-06-26

**Authors:** Rafał Staszkiewicz, Dawid Sobański, Wojciech Pulka, Dorian Gładysz, Marcin Gadzieliński, Damian Strojny, Beniamin Oskar Grabarek

**Affiliations:** 1 Collegium Medicum, WSB University, 41-300 Dabrowa Gornicza, Poland; drdsobanski@gmail.com (D.S.); gladyszdorian875@gmail.com (D.G.); gadzielinskimarcin@gmail.com (M.G.); strojny.ds@gmail.com (D.S.); bgrabarek7@gmail.com (B.O.G.); 2 Department of Neurosurgery, 5th Military Clinical Hospital with the SP ZOZ Polyclinic in Krakow, 30-901 Cracow, Poland; 3 Department of Neurosurgery, Faculty of Medicine in Zabrze, Academy of Silesia, 40-555 Katowice, Poland; 4 Department of Neurosurgery, Szpital sw. Rafala in Cracow, 30-693 Cracow, Poland; 5 Department of Neurosurgery, Neurotraumatology and Spinal Surgery, Regional Hospital in Elblag, 82-300 Elblag, Poland; ns.wojciechpulka@gmail.com; 6 Institute of Health Care, National Academy of Applied Sciences in Przemysl, 37-700 Przemysl, Poland; 7 New Medical Techniques Specjalist Hospital of St. Family in Rudna Mała, 36-054 Rudna Mala, Poland

**Keywords:** circadian clocks, brain neoplasms, astrocytic tumor, RNA, messenger, microRNAs, enzyme-linked immunosorbent assay, biomarkers

## Abstract

**Simple Summary:**

This study investigates the role of circadian clock genes in the progression of astrocytic tumors, a common type of brain tumor. We aimed to understand how these genes, which control the body’s daily rhythms, behave differently in low-grade versus high-grade tumors. Our findings reveal that certain circadian clock genes are more active in advanced tumor stages, potentially driving tumor growth. Additionally, we discovered that changes in DNA methylation and microRNAs might regulate these genes. Understanding these molecular changes could help identify new biomarkers for tumor diagnosis and progression, offering new avenues for targeted treatments. This research provides valuable insights into the complex biology of brain tumors and highlights the importance of circadian genes in the development of cancer.

**Abstract:**

This study explores the role of circadian clock genes in the progression of astrocytic tumors, a prevalent type of brain tumor. The aim was to assess the expression patterns of these genes in relation to the tumor grade. Using microarray analysis, qRT-PCR, and methylation-specific PCR, we examined gene expression, DNA methylation patterns, and microRNA interactions in tumor samples from 60 patients. Our results indicate that the expression of key circadian clock genes, such as clock circadian regulator *(CLOCK)*, protein kinase AMP-activated catalytic subunit alpha 1 *(PRKAA1)*, protein kinase AMP-activated catalytic subunit alpha 2 *(PRKAA2)*, protein kinase AMP-activated non-catalytic subunit beta 1 *(PRKAB1)*, protein kinase AMP-activated non-catalytic subunit beta 2 *(PRKAB2)*, period circadian regulator 1 *(PER1)*, period circadian regulator 2 (*PER2*) and period circadian regulator 3 *(PER3)*, varies significantly with the tumor grade. Notably, increased CLOCK gene expression and protein levels were observed in higher-grade tumors. DNA methylation analysis revealed that the promoter regions of PER1-3 genes were consistently methylated, suggesting a mechanism for their reduced expression. Our findings also underscore the complex regulatory mechanisms involving miRNAs, such as hsa-miR-106-5p, hsa-miR-20b-5p, and hsa-miR-30d-3p, which impact the expression of circadian clock-related genes. This underscores the importance of circadian clock genes in astrocytic tumor progression and highlights their potential as biomarkers and therapeutic targets. Further research is needed to validate these results and explore their clinical implications.

## 1. Introduction

Astrocytic tumors (tumors originating from astrocytes) are among the most common brain tumors and are categorized into four grades based on their characteristics and severity [1,2,3]. Despite significant advances in clinical research, the prognosis for these diseases remains poor. Patients with low-grade gliomas (LGGs) (grades II and III) have an average survival time of 5–10 years, while those with high-grade gliomas (grade IV) typically survive for 1–2 years [4]. Glioblastoma multiforme (GBM), a grade IV astrocytic tumor, is particularly prevalent and highly malignant [5,6]. Advancements in molecular biology have led to a revised brain tumor classification by the World Health Organization (WHO), released in 2016, which incorporates genome characterization and the identification of epigenetic changes [7]. The latest classification of central nervous system tumors, implemented in 2021, underscores the growing significance of molecular biology methods in diagnosing and classifying brain tumors. The 2021 WHO classification introduced a new entity: astrocytoma grade IV, to distinguish it from the more common glioblastoma (also grade IV). Some tumors previously classified as grade III astrocytomas have now been defined as GBM IV under the new classification [8,9,10]. Molecular biomarkers provide crucial diagnostic information, sometimes being the sole identifiers of specific tumors, while in other cases they offer supplementary data [11].

Several markers are critical for predicting prognosis and treatment response, including isocitrate dehydrogenase 1/2 (IDH) mutation status, MGMT promoter methylation status, co-deletion of chromosome arms 1p and 19q, and epidermal growth factor receptor (EGFR) amplification [12,13,14]. Specifically, IDH mutations are associated with a better prognosis across all glioma grades. For instance, patients with IDH-mutated grade IV astrocytic tumors have a median survival of 31 months, compared to 15 months for patients with grade IV IDH wild-type astrocytic tumors [12,13,14].

The methylation profile of tumors plays a vital role in accurately identifying nearly all brain tumors, and it is now used alongside traditional histological methods in diagnosis. Integrating traditional histological and molecular techniques is also expected to enhance prognostic assessments of brain tumors [15]. It is considered appropriate to classify a tumor as malignant if characteristic molecular alterations are present, even if histological examination suggests a lower grade of malignancy [8,16]. 

Many cellular processes involved in gliomagenesis and progression are also regulated by the circadian clock (from the Latin circa (near)/dies (day)) genes, which exhibit daily rhythms (24-hour cycles) of expression in healthy tissues. Circadian rhythms influence cellular functions through a transcriptional–translational feedback loop (TTFL) involving core clock genes, including the transcriptional activators CLOCK and ARNTL and the transcriptional repressors PER1/2/3 and CRY1/2. In the brain’s central pacemaker, the suprachiasmatic nucleus (SCN), *PER* gene expression is synchronized and triggered by exposure to light. Once transcribed, PER and CRY proteins move into the nucleus to interact with CLOCK and ARNTL, suppressing the expression of PER and CRY. Additional TTFLs involve components such as RORα/β/γ, REV-ERBα/β, DBP, TEF, and HLF, which complement the core clock and ensure antiphase oscillation of ARNTL relative to PER and CRY. These feedback loops create rhythmic fluctuations in the transcriptome and modulate cellular processes by regulating clock-controlled genes (CCGs). The expression of clock genes and regulation of CCGs vary depending on the tissue and cell types [17,18,19].

In various cancers, disruption of the biological clock by environmental factors or mutations in the circadian pathway can increase the risk of tumorigenesis. Deregulated circadian clock genes are implicated in astrocytic tumors. High-grade astrocytic tumors show significantly enhanced expression levels of core circadian clock genes compared to low-grade astrocytic tumors and non-tumor tissues. Differential expression patterns of clock genes in astrocytic tumor cells versus adjacent normal brain tissues indicate circadian clock asynchrony [20,21,22,23]. This finding was supported by another study using SNP array analysis, which identified chromosomal number alterations—specifically, amplification at the 4q12 chromosomal region, where the mammalian *CLOCK* gene is located. The DNA copy number alteration affected mRNA levels, suggesting a strong correlation with disease pathogenesis [24].

Therefore, the goal of our research was to assess differences in the expression patterns of genes associated with the circadian clock in astrocytic tumors with respect to the degree of malignancy.

## 2. Materials and Methods

### 2.1. Ethics

Ethical considerations were a primary focus in this study, strictly following the guidelines of the 2013 Declaration of Helsinki related to human experimentation. The Bioethical Committee of the Regional Medical Chamber in Krakow granted approval, with reference number 247/KBL/OIL/2021, dated 11 June 2021. Throughout the study, rigorous measures were implemented to protect data confidentiality and maintain patient anonymity. All identifying information was carefully removed from the database before analysis, ensuring that individual patient identification was not possible within this article or the database itself. 

### 2.2. Participants

Specimens of astrocytic sections of brain tumors were collected from 60 patients. The diagnosis of a brain tumor, qualification for surgery, and resection of the lesion were performed at the Department of Neurosurgery, 5th Military Clinical Hospital with the SP ZOZ Polyclinic in Krakow, Poland and the Department of Neurosurgery, Szpital sw. Rafala, Krakow, Poland.

The initial diagnosis of astrocytic tumors in the patients was established via contrast-enhanced computed tomography. This was followed by magnetic resonance imaging (MRI) with and without contrast, using T1- and T2-weighted sequences, fluid-attenuated inversion recovery sequences (which are a modification of the T2-weighted sequence), and/or diffusion tensor imaging. In the case of tumor localization near the eloquent areas of the brain, the aforementioned basic MRI sequences were extended to functional MRI and diffusion MRI tractography to use them in the neuronavigation system.

During surgery, surgical resection was performed as widely as possible using neuronavigation, fluorescence imaging with 5-aminolevulinic acid (5-ALA; only in the IV G4 stage), and, for tumors located near the sensorimotor cortex, electrical stimulation of the brain. The final diagnosis was confirmed by histopathological evaluation of the resected lesion. Based on the histopathological examination, tumor malignancy grades were determined according to the WHO scale: grade II (G2), grade III (G3), and grade IV (G4) (Table 1).

Patient eligibility for the study was determined based on specific inclusion and exclusion criteria. The inclusion criteria required patients to be hospitalized in the Department of Neurosurgery, 5th Military Clinical Hospital with the SP ZOZ Polyclinic in Krakow, Poland or the Department of Neurosurgery, Szpital sw. Rafala, Krakow, Poland and qualified for surgery for an astrocytic brain tumor. Additionally, the patients had to provide informed consent to participate in the study. The exclusion criteria specified that patients who had another neoplastic lesion were not eligible for the study. 

All patients included in the study group consumed their last meal (dinner) at 6 p.m. and fasted from that point on (14–17 h). In the hospitals where the study was conducted, nutritional policy provides the last meal around 6 p.m., and the next day the patients undergo surgery. Surgery for all patients took place during the same morning hours (8–11); only elective patients were included.

### 2.3. Isolation of Total Ribonucleic Acid (RNA)

Samples were homogenized using a handheld rotor/stator homogenizer (T18 Digital Ultra-Turrax, IKA Poland Ltd., Warsaw, Poland). Thereafter, total RNA extraction from tissues began with TRIzol reagent (Invitrogen Life Technologies, Carlsbad, CA, USA; Catalog number: 15596026), strictly adhering to the manufacturer’s instructions. Following this, the RNeasy mini kit (QIAGEN, Hilden, Germany; Catalog number: 74104) was utilized to purify the isolated RNA, effectively removing any impurities and contaminants. To further refine the RNA samples, DNase I treatment (Fermentas International Inc., Burlington, ON, Canada; Catalog number: 18047019) was applied to eliminate any residual genomic DNA.

The quality of the extracted RNA was assessed using 1% agarose gel electrophoresis with 0.5 mg/mL ethidium bromide, allowing for the visualization and evaluation of RNA integrity. Additionally, RNA concentrations were quantified by measuring absorbance at 260 nm, providing an accurate measure of the yield and purity of the RNA samples.

### 2.4. Microarray Expression Pattern of Circadian Clock-Related Genes

The list of genes associated with circadian clock-related genes was compiled using data from the Kyoto Encyclopedia of Genes and Genomes (KEGG database; http://pathcards.genecards.org/, accessed on 4 May 2024) [25].

A comparative expression analysis of circadian clock-related genes in tumor tissues versus control tissues was performed using the HG-U 133_A2 microarray platform (Affymetrix, Santa Clara, CA, USA) and the GeneChip™ 3′ IVT PLUS reagent kit (Affymetrix; Catalog Number 902416), strictly following the manufacturer’s protocols and methodologies from previous studies. The microarray analysis protocol included the synthesis of double-stranded complementary DNA (cDNA) using the GeneChip 30IVT Express kit, followed by RNA amplification and fragmentation. The amplified RNAs (aRNAs) were then hybridized, and fluorescence intensity was measured using an Affymetrix Gene Array Scanner 3000 7G along with the Gene Chip^®^ Command Console^®^ Software 6.0+ (Affymetrix). This comprehensive approach enabled a detailed examination of differential gene expression patterns within the histaminergic system, highlighting potential molecular changes associated with tumorigenesis.

### 2.5. Comprehensive Microarray Profiling of Circadian Clock-Related miRNAs and Their Potential Impact on Gene Expression

To explore the complex role of circadian clock-related miRNAs and their potential impact on gene expression, we conducted a microarray analysis using the GeneChip miRNA 2.0 Array (Affymetrix), a highly reliable and precise commercial platform. The microarray profiling procedure strictly followed the manufacturer’s instructions to ensure standardized and reproducible results. Differentially expressed miRNAs between tumor and control tissues, which are crucial for modulating the expression of differentially expressed mRNAs, were meticulously identified using two reputable databases: TargetScan (http://www.targetscan.org/, accessed on 20 May 2023) [26] and miRanda (http://mirdb.org, accessed on 20 May 2023) [27]. These databases provide valuable insights into potential miRNA–mRNA interactions, aiding in elucidating the underlying regulatory mechanisms [27,28]. In our analysis, a predicted target with a prediction score exceeding 80 was considered highly credible, indicating a strong miRNA–mRNA interaction. However, it is essential to exercise caution when interpreting results with prediction scores below 60, as these may require additional corroborating evidence to validate their authenticity [27,28]. 

### 2.6. Quantitative Reverse-Transcription Polymerase Chain Reaction (qRT-PCR) Analysis

To validate the microarray data, qRT-PCR was performed on selected genes using the SensiFast SYBR No-ROX One-Step kit (Bioline, London, UK), following the manufacturer’s guidelines. The thermal conditions for the one-step RT-qPCR were as follows: reverse transcription at 45 °C, polymerase activation at 95 °C for 2 min, followed by 40 duplicate cycles of denaturation at 95 °C for 5 s, annealing at 60 °C for 10 s, and final elongation at 72 °C for 5 s. Gene expression profiles were analyzed using the 2^−ΔΔCt^ method, where a fold change of 1 represented the control, values greater than 1 indicated overexpression, and values less than 1 indicated gene silencing. β-Actin (ACTB) served as the internal control for normalization. Detailed primer sequences are provided in Table 2 for reference. 

### 2.7. Methylation Analysis of Genes Using PCR

In the initial phase, we identified the locations of CpG islands within the sequences. Using the MethPrimer program (http://www.urogene.org/cgi-bin/methprimer/methprimer.cgi; accessed on 4 May 2024), primers were designed to detect methylated and non-methylated sequences via PCR, with the following criteria: CpG island length of >100 nucleotides, >50% GC content, and an observed-to-expected ratio of >0.6 (Table 3).

To assess gene methylation, bisulfite conversion was performed using sodium bisulfite according to the manufacturer’s recommendations, followed by purification of the extract. Methylation-specific PCR (MSP) was then conducted using the QuantiTect SYBR Green PCR Kit (Qiagen GmbH, Hilden, Germany) and the primers listed in Table 4. The thermal cycling profile was as follows: initial denaturation at 95 °C for 5 min, followed by 40 cycles of 30 s each at 94 °C (denaturation), 65 °C (primer annealing), and 72 °C (elongation).

Following the amplification of selected genes, the PCR products were separated by electrophoresis on 1% agarose gel containing ethidium bromide (final concentration 0.5 µg/mL) in 1× TBE buffer at 120 V. Fragment analysis was performed using the pBR322/HaeIII size marker. The accuracy of amplification was verified with the positive control (methylated DNA) and negative control (non-methylated DNA) using the EpiTect Control DNA (Qiagen GmbH, Hilden, Germany) set.

### 2.8. Enzyme-Linked Immunosorbent Assay (ELISA) Reaction

The concentrations of selected genes in cancerous tissues were determined using ELISA kits: the Human Period Circadian Protein 1 (PER1) ELISA Kit (MyBioSource, Inc., San Diego, CA, USA, catalog number MBS2020223); Protein Kinase AMP Activated Alpha 1 (PRKAa1) ELISA Kit (MyBioSource, Inc., San Diego, CA, USA, catalog number MBS2024096); 5′-AMP-activated protein kinase catalytic subunit alpha-2 ELISA Kit (MyBioSource, Inc., San Diego, CA, USA, catalog number MBS760387); 5′-AMP-activated protein kinase subunit beta-1 ELISA Kit (MyBioSource, Inc., San Diego, CA, USA, catalog number MBS762547); Protein Kinase AMP Activated Beta 2 (PRKAb2) ELISA Kit (MyBioSource, Inc., San Diego, CA, USA, catalog number MBS2127347); Period Circadian Protein 1 (PER1) ELISA Kit (MyBioSource, Inc., San Diego, CA, USA, catalog number MBS2020223); Period Circadian Protein 2 (PER2) ELISA Kit (MyBioSource, Inc., San Diego, CA, USA, catalog number MBS4504464); and PER3/Period Circadian Protein Homolog 3 ELISA Kit (MyBioSource, Inc., San Diego, CA, USA, catalog number MBS2890747).

The assays were performed according to the manufacturer’s instructions. Each biological replicate was tested in three technical repetitions. Absorbance was measured at 540 nm using an Infinite M200 PRO microplate reader (Tecan, Männedorf, Switzerland).

### 2.9. Statistical Analyses

Statistical analyses were performed using licensed versions of Statistica 13.0 PL (StatSoft, Cracow, Poland) and the Transcriptome Analysis Console programs (Affymetrix). The Shapiro–Wilk test was used to assess the normality of data distribution, with significance set at *p* < 0.05. Mean differences were analyzed via analysis of variance (ANOVA) with Benjamini–Hochberg correction, followed by Tukey’s post hoc test (*p* < 0.05) or Student’s *t*-test, depending on the specific comparison. 

The relationships between genes were examined using the Search Tool for the Retrieval of Interacting Genes/Proteins (STRING Database 11.0; accessed 5 May 2024). Within the STRING database, the parameter strength Log10 (observed/expected) quantifies the extent of the enrichment effect; it reflects the ratio of (1) the number of proteins annotated with a specific term within the network to (2) the expected number of proteins annotated with that term in a randomly generated network of equivalent size. The false discovery rate parameter assesses the significance of the enrichment, with *p*-values adjusted for multiple testing within each category using the Benjamini–Hochberg procedure [29].

## 3. Results

### 3.1. Microarray and qRT-PCR Profile of Circadian Clock-Related Genes in G3/G4 Samples of Astrocytic Tumors in Comparison to G2 Samples

**Figure 1:**
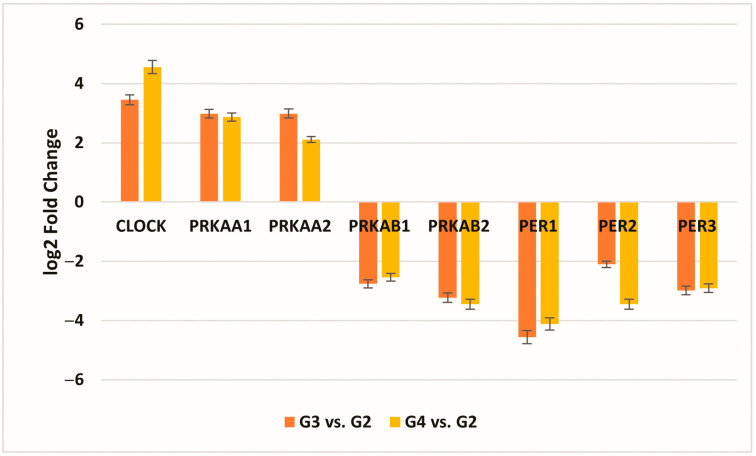
Changes in the expression profile of genes differentiating tumor samples compared to control tissues obtained by the microarray analysis (−2.0 < FC > 2.0; *p* < 0.05). (*CLOCK*, clock circadian regulator; *PRKAA1*, protein kinase AMP-activated catalytic subunit alpha 1; *PRKAA2*, protein kinase AMP-activated catalytic subunit alpha 2; *PRKAB1*, protein kinase AMP-activated non-catalytic subunit beta 1; *PRKAB2*, protein kinase AMP-activated non-catalytic subunit beta 2; *PER1*, period circadian regulator 1; *PER2*, period circadian regulator 2; *PER3*, period circadian regulator 3; FC, fold change; G2—II grade; G3—III grade; G4—IV grade. Data are presented as the mean ± standard deviation).

In the statistical evaluation of the microarray analysis, out of the 34 circadian clock-related mRNAs compiled using the KEGG database, a one-way ANOVA test showed that 8 mRNAs were significantly changed in G3/G4 samples in comparison to G2 samples (−2.0 < FC > 2.0; *p* < 0.05), of which 3 genes were common to the G3 and G4 stages compared to the G2 stage: *CLOCK*, *PRKAA1*, and *PRKAA2* (Figure 1).

**Figure 2:**
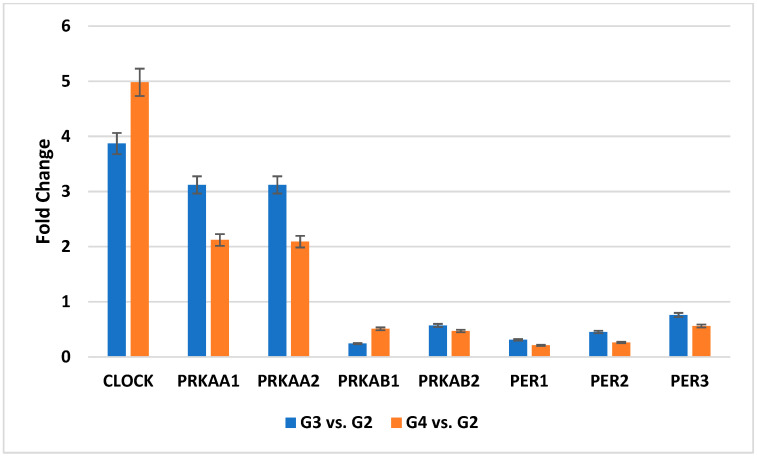
Variances in the expression of selected genes related to the circadian clock obtained by the qRT-PCR. (*CLOCK*, clock circadian regulator; *PRKAA1*, protein kinase AMP-activated catalytic subunit alpha 1; *PRKAA2*, protein kinase AMP-activated catalytic subunit alpha 2; *PRKAB1*, protein kinase AMP-activated non-catalytic subunit beta 1; *PRKAB2*, protein kinase AMP-activated non-catalytic subunit beta 2; *PER1*, period circadian regulator 1; *PER2*, period circadian regulator 2; *PER3*, period circadian regulator 3; FC, fold change; G2—II grade; G3—III grade; G4—IV grade).

The recorded microarray pattern of transcriptional activity of the genes assessed was confirmed by qRT-PCR (Figure 2).

### 3.2. Prediction of Circadian Clock Gene Expression Regulation by miRNAs

**Figure 3:**
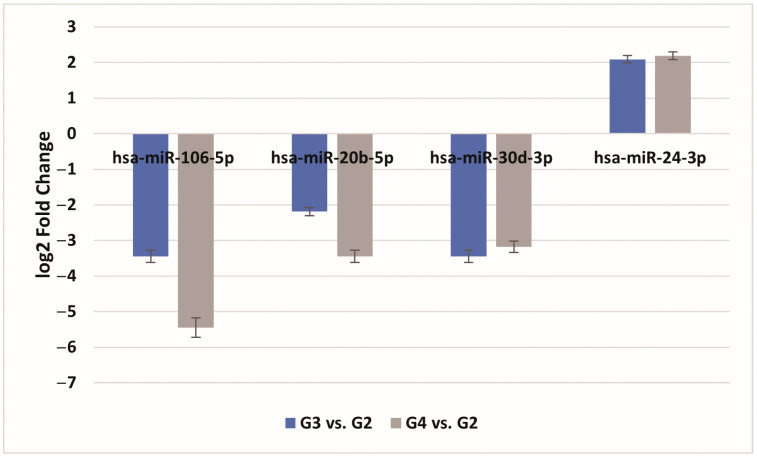
The expression profile of miRNAs potentially regulated by selected mRNAs in G3/G4 samples of astrocytic tumors in comparison to G2 samples; FC, fold change. Data are presented as the mean ± standard deviation. G2—II grade; G3—III grade; G4—IV grade).

We further investigated whether the expression of genes that distinguish astrocytic tumor cancer samples, regardless of subtype, could be regulated by miRNA molecules. The predictive analysis indicated that hsa-miR-106-5p (target score 99) and hsa-miR-20b-5p (target score 99) can regulate the expression of *CLOCK*, while hsa-miR-30d (target score 99) is potentially involved in regulating *PRKAA2* expression. Additionally, the expression patterns of *PER1* and *PER2* mRNA can be regulated by hsa-miR-24-3p (target score 87). Conversely, the predictive analysis did not show that the expression of *PRKAA1*, *PRKAB1*, *PRKAB2*, and *PER3* is regulated by miRNAs in astrocytic tumors (Figure 3; *p* < 0.05).

### 3.3. Methylation Profile of Selected Genes Related to the Circadian Clock in Astrocytic Tumor Samples

**Figure 4:**
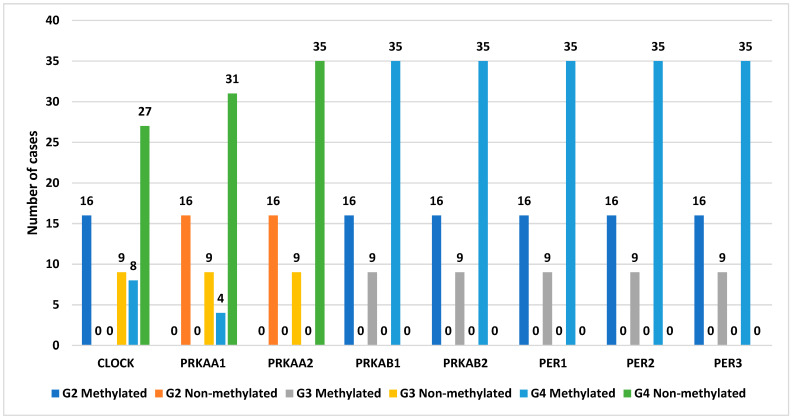
The degree of methylation of selected genes in the G2, G3, and G4 astrocytic tumor samples. (*CLOCK*, clock circadian regulator; *PRKAA1*, protein kinase AMP-activated catalytic subunit alpha 1; *PRKAA2*, protein kinase AMP-activated catalytic subunit alpha 2; *PRKAB1*, protein kinase AMP-activated non-catalytic subunit beta 1; *PRKAB2*, protein kinase AMP-activated non-catalytic subunit beta 2; *PER1*, period circadian regulator 1; *PER2*, period circadian regulator 2; *PER3*, period circadian regulator 3; G2—II grade; G3—III grade; G4—IV grade).

For the *CLOCK* gene, we observed that all samples in the G2 and G3 stages were methylated. In contrast, in the G4 stage, the methylation pattern was provided for only eight samples. The same methylation pattern was noted for *PRKAA1*. Conversely, methylation was confirmed in all samples for *PRKAA2*. Additionally, for the genes *PRKAB1*, *PRKAB2*, *PER1*, *PER2*, and *PER3*, methylation was observed in all samples, regardless of the tumor grade. The degree of methylation in the G2, G3, and G4 samples is presented in Figure 4.

### 3.4. Concentration of Selected Proteins Related to the Circadian Clock in the G2, G3, and G4 Astrocytic Tumor Samples

As a final step, we assessed the concentrations of proteins encoded by the genes discussed above in astrocytic brain tumors at stages G2, G3, and G4 (Table 4; *p* < 0.05). Instead of the concentrations of PER1-3, the lowest concentrations of the analyzed proteins were found in samples representing stage G2. In turn, for PER1–3, the highest concentrations were observed for samples representing G2, while the lowest were observed for G4 samples. Detailed changes in the concentration profiles of the selected proteins are shown in Table 4 (*p* < 0.05).

### 3.5. Relationship Network for the Selected Circadian Clock-Related Proteins 

**Figure 5:**
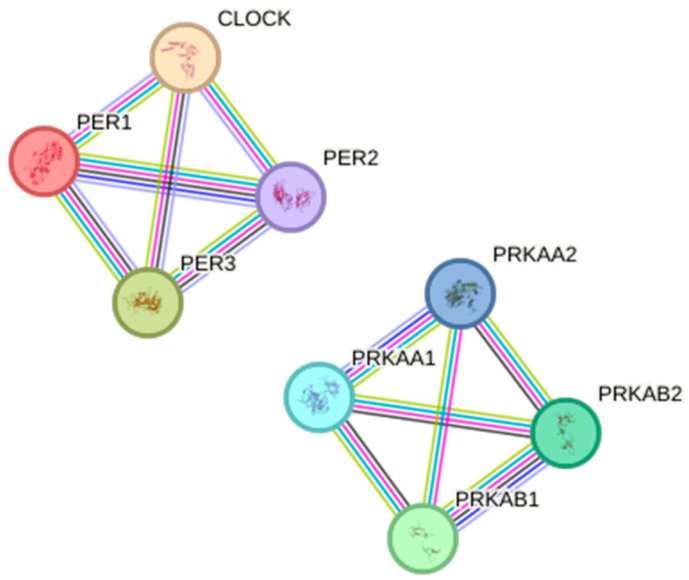
Relationship network for the selected circadian lock differentiation proteins generated in the STRING database. (CLOCK, clock circadian regulator; PRKAA1, protein kinase AMP-activated catalytic subunit alpha 1; PRKAA2, protein kinase AMP-activated catalytic subunit alpha 2; PRKAB1, protein kinase AMP-activated non-catalytic subunit beta 1; PRKAB2, protein kinase AMP-activated non-catalytic subunit beta 2; PER1, period circadian regulator 1; PER2, period circadian regulator 2; PER3, period circadian regulator 3. The green line for interaction indicates “textmining”, the black line “co-expression”, the turquoise line “interactions from curated databases”, the pink line “interactions experimentally determined”, the blue line “gene co-occurrence”, and the violet line “protein homology”).

The network diagram shows interactions between proteins involved in circadian rhythms (CLOCK, PER1, PER2, PER3) and energy metabolism (PRKAA1, PRKAA2, PRKAB1, PRKAB2), generated using the STRING database (version 11.0). Each node represents a protein encoded by the examined genes, and each edge represents a potential protein–protein interaction, with the edge thickness indicating the strength of the interaction. The network consists of 12 nodes and eight edges, with a high average local clustering coefficient of 1.0 and an average node degree of 3.0, indicating a highly interconnected network (Figure 5; *p* < 0.0001). The left cluster includes CLOCK and the PER proteins, indicating their close interaction in regulating circadian rhythms.

The right cluster includes the PRKAA and PRKAB subunits, indicating their interaction within the AMPK complex to regulate energy homeostasis. The color-coded lines between the proteins represent different types of interactions, such as physical binding, co-expression, or functional association. The dense interconnections among PER1, PER2, PER3, and CLOCK suggest a tightly regulated network essential for maintaining circadian rhythms. Similarly, the interconnections among the AMPK subunits indicate a cohesive regulatory mechanism for energy balance. The circadian rhythm proteins and AMPK proteins might interact to coordinate the body’s energy needs with its daily activity cycles (Figure 5). 

## 4. Discussion

Brain tumors constitute a diverse group due to the varying morphology of the cells from which they originate. In 2007, the World Health Organization (WHO) categorized these tumors into four grades based on histological malignancy [30]. Advances in molecular biology have facilitated genome characterization and the identification of epigenetic changes. This progress led to a new classification in 2016, which incorporated gene analysis alongside histological criteria for a more precise categorization of these cancers [7]. This classification was further updated in 2021 [8,9]. The differences in histology, symptoms, and gene expression profiles between low- and high-grade gliomas underscore the importance of molecular studies in clinical diagnosis and treatment [8,9]. Recent advancements in molecular biology have provided deeper insights into the genetic and epigenetic landscapes of these tumors, leading to refined classifications and improved diagnostic capabilities [31]. 

Research performed by Marko et al. on primary glioblastoma multiforme (GBM) identified 1473 genes, with at least 43 showing significant differences in expression levels correlating with patient survival times; additionally, a correlation was found between tumor genotype and patient survival duration [32].

Circadian clock genes have emerged as significant players among the various molecular factors implicated in tumor progression. These genes, which govern the circadian rhythms of physiological processes through intricate transcriptional–translational feedback loops, have shown altered expression patterns in various cancers, including astrocytic tumors [17,18,19]. In the present study, we aimed to assess changes in the expression patterns of mRNAs encoding circadian clock-related proteins using two molecular biology methods: mRNA microarrays, and qRT-PCR. Subsequently, we investigated whether the expression of genes identified in the microarray experiment could be regulated by miRNA molecules, evaluating the strength of these interactions and their translation into protein concentrations. Finally, we analyzed whether DNA methylation influenced the evaluated genes’ expression profiles. 

*CLOCK* mRNA was the first of the transcripts differentiating astrocytic brain tumor sections at stage G3/G4 vs. G2. We found significantly higher expression in more advanced tumor stages at the mRNA and protein levels. Moreover, Li et al., using the Western blot technique, noted an increase in the CLOCK protein concentration in tumor stages G3 and G4 [33]. Additionally, Chen et al. found an increase in the transcriptional activity of the *CLOCK* gene using the qRT-PCR technique [20]. This indicates that the *CLOCK* gene and the protein that it encodes may be among the important factors of tumor progression. However, the roles of CLOCK in the generation and progression of gliomas are not well understood. Nevertheless, Li et al. indicated that the CLOCK gene and the protein encoded by it contribute to the progression of astrocytic tumors by influencing the nuclear factor kappa-B (NFκB) signaling pathway and the expression of miR-124 [33]. 

CLOCK and BMAL1 (also known as ARNTL) are essential transcription factors in the circadian system, forming a heterodimeric complex. This complex activates the expression of PER and CRY genes, which then create a negative feedback loop that inhibits the activity of the CLOCK–BMAL1 complex [34]. 

There is growing recognition that the roles of CLOCK and BMAL1 in the development of cancer vary significantly depending on the context and type of disease [35]. For instance, CLOCK or BMAL1 can act as a tumor suppressor in prostate, breast, ovarian, and pancreatic cancers, but they promote tumor growth in colorectal cancer and acute myeloid leukemia [35,36]. In glioblastoma (GBM), CLOCK or BMAL1 can promote tumor growth by regulating glioma cell proliferation and migration through the NFκB pathway and supporting glioma stem-cell function by managing anabolic metabolism [33,37]. 

Furthermore, Pan et al. indicated that overexpression of the CLOCK gene contributes to the progression of astrocytic brain tumors, among others, by exerting a promoting effect on the process of tumor angiogenesis and regulating the olfactomedin-like 3 (OLFML3) system—hypoxia-inducible factor 1-alpha (HIF1a)–transcriptional upregulation of periostin (POSTN)–TANK-binding kinase 1 (TBK1) [38]—and activating the canonical SMAD1/5/8 signaling pathway [39]. POSTN, also known as osteoblast-specific factor, is a multifunctional matricellular protein initially identified in osteoblasts; it plays a significant role in regulating inflammatory responses and the tumor microenvironment (TME) [40]. POSTN is crucial for the migration of macrophages and epithelial cells into the TME through its interaction with αVβ3 and αVβ5 integrins [41]. Research has shown that TBK1 is a key downstream signaling molecule responsible for POSTN-induced angiogenesis in GBM. TBK1 is vital for type I interferon production during antiviral immune responses and acts as an oncogene in cancer cells, regulating cell division, autophagy, and AKT pro-survival signaling in melanoma and lung cancer. In glioblastoma stem cells (GSCs) [42,43], TBK1 suppresses the core pluripotency circuitry. Furthermore, TBK1’s role in promoting tumor angiogenesis is supported by studies indicating that TBK1 in cancer cells enhances angiogenesis through a non-cell-autonomous mechanism by upregulating vascular endothelial growth factor (VEGF) [44]. 

A characteristic feature of the GBM TME is the presence of numerous infiltrating immune cells, with microglia playing a key role in creating an immunosuppressive environment that supports GBM’s progression [22,45]. Circadian components can regulate the immune system, and disruption of the intrinsic circadian clock can modify inflammatory responses [46,47]. Notably, microglia express OLFML3, suggesting that after being recruited by CLOCK-directed mechanisms, they might further amplify the recruitment of additional microglia by secreting OLFML3 in a self-perpetuating cycle [48,49]. In addition, Wang et al. confirmed that samples with a higher risk score exhibited a greater infiltration of T cells, NK cells, macrophages, and dendritic cells compared to those with a lower risk score. This indicates that high-risk score tumors tend to exhibit a “hot” tumor phenotype, suggesting a higher likelihood of sensitivity to immunotherapy [22]. Additionally, other studies have demonstrated a strong association between disrupted circadian rhythms and the components of the tumor microenvironment, immune cell activation, and responses to immunotherapy [50,51].

Nevertheless, to the best of the authors’ knowledge, the present study is the first to evaluate differences in the *CLOCK* methylation patterns in cerebral stromal tumors. Therefore, the methylation pattern of the *CLOCK* gene plays an important role in the pathogenesis of astrocytic tumors. Both hypermethylation and hypomethylation can disrupt its normal function, leading to altered circadian regulation, increased cell proliferation, and tumor progression. Understanding these epigenetic changes provides valuable insights into astrocytic tumors’ biology and opens up potential opportunities for prognostic biomarkers and targeted therapies. 

In a subsequent step, we determined that CLOCK mRNA expression can also be regulated at the post-transcriptional level via miRNA molecules, as well as titers of hsa-miR-106-5p and hsa-miR-20b-5p, whose expression statistically significantly decreases with increasing tumor grade. It is also important to emphasize the strong interaction between CLOCK mRNA and the two target miRNAs (Target score 99). Nevertheless, CLOCK protein levels are higher at higher tumor lesion stages (G2 > G3 > G4). 

Hsa-miR-106b-5p is part of the 106b-25 cluster and a paralog of the 17–92 cluster. Preclinical studies have demonstrated that hsa-miR-106b-5p can predict metastasis. Hsa-miR-106b-5p has been shown to promote astrocytic tumor-cell proliferation; by targeting tumor suppressor genes, it facilitates unchecked cell division, contributing to tumor growth [52]. A decrease in miR-106a levels has been linked to shorter disease-free and overall survival in patients with human colon cancer [53]. Hang et al. provided crucial evidence that low expression of miR-106a is significantly correlated with the malignant progression of human astrocytic tumors [54]. Additionally, recent studies found that low miR-106a expression was associated with poor patient survival in 84 astrocytoma samples [23,55]. Zhao et al. reported that miR-106a exhibits a 13-fold lower mean expression level in GBM samples compared to normal brain tissues [56]. Although the function of miR-106a in the genesis and progression of tumors is not fully understood, previous research has demonstrated that miR-106a exerts a tumor-suppressive effect by inhibiting proliferation and inducing apoptosis in human astrocytic tumor cells. This suppressive effect may result from the inhibition of E2F1 via post-transcriptional regulation [57,58]. 

Interestingly, miR-20b, located within 1 kb of miR-106a, has been identified as a downregulated miRNA that inhibits Th17 differentiation and mitigates the severity of the autoimmune inflammatory response by targeting RORγt and STAT3. Hong et al. discovered that miR-20b acts as a tumor suppressor in thyroid cancer by modulating the MAPK/ERK signaling pathway [59]. Zhou et al. found that miR-20b promoted the proliferation of breast cancer cells by modulating PTEN [60]. Additionally, Zhou et al. found that miR-20b promotes the proliferation of breast cancer cells by regulating PTEN. In glioma studies, Zhou et al. observed that the expression of miR-106a and miR-20b was downregulated in dendritic cells pulsed with glioma stem cells [52]. Furthermore, Huang et al. confirmed the reduced expression of miR-20b in glioma samples [61].

Further circadian clock-related genes whose expression was statistically significantly altered in the samples were *PRKAA1*, *PRKAA2*, *PRKAB1*, and *PRKAB2*. These mRNAs encode the catalytic alpha subunits of AMP-activated protein kinase (AMPK), a crucial energy sensor and regulator of cellular metabolism. The AMPK pathway plays a vital role in maintaining cellular energy homeostasis by activating catabolic processes that generate ATP while inhibiting anabolic processes that consume ATP. Dysregulation of AMPK signaling has been implicated in various diseases, including cancer [62,63,64]. Recent studies have highlighted the significance of PRKAA1 and PRKAA2 in tumorigenesis and cancer progression. Alterations in the expression and activity of these genes have been observed in several types of cancer. For instance, PRKAA1 is overexpressed in certain malignancies, contributing to enhanced cell survival and proliferation under metabolic stress conditions [65,66]. PRKAA2 is crucial for both the initiation and progression of tumors; it is involved in regulating mTOR kinase activity and maintaining NADPH levels [55]. Variations in PRKAA2 expression are associated with the onset, development, and prognosis of several cancers, such as breast, ovarian, gastric, kidney, and liver hepatocellular carcinoma [67,68]. Therefore, PRKAA2 emerges as a promising target for novel therapeutic approaches and may also impact tumor immunity in certain cancer types [55]. Our analysis of the mRNA expression patterns of PRKAA1 and PRKAA2, along with their encoded proteins, revealed that their expression increases with the grade of the astrocytic tumor. Additionally, our findings underscore the significant role of DNA methylation and miRNA molecules in regulating these genes. The study by Ouyang et al. highlighted that PRKAA2 facilitates immune escape in tumor cells by reducing CD8+ T cells and promoting the generation of regulatory T cells (Tregs), thereby contributing to tumor progression [68]. Moreover, malignant cells with high PRKAA2 expression evade immune suppression by losing major histocompatibility complex class I (MHC-I)-mediated interferon-gamma/Janus kinase/signal transducer and activator of transcription (IFN-γ/JAK/STAT) pathway molecules [69]. On the other hand, Varghese et al. indicated that reduced PRKAA1 expression is a non-correlated prognostic factor in glioblastomas, highlighting the heterogeneity of the tumor cells, which probably influences the results obtained [70]. The obtained expression patterns of *PRKAA1* and *PRKAA2* mRNA, along with the proteins that they encode, indicate the induction of hypoxia in the tumor microenvironment [71]. Moreover, we found that hsa-miR-30d can affect the expression profile of *PRKAA2.* It has been indicated that restoring the normal expression pattern of hsa-miR-30d-3p may be a promising anticancer strategy [72,73]. In addition, it has also been suggested that hsa-miR-30d-3p is involved in hypoxia in neurodegenerative, ischemic, and cancerous diseases [74,75]. 

The transcriptional activity of the *PRKAB1* and *PRKAB2* genes significantly decreases as the grade of astrocytic brain tumors increases. This decline is also evident at the protein level, with the lowest levels of both proteins observed in G4 astrocytic tumors. As indicated by studies conducted on various types of cancer, lower expression of PRKAB1 and PRKAB2 resulted in increased cell proliferation and enhanced metastatic potential of tumors. This suggests that PRKAB1 and PRKAB2 function as tumor suppressor genes [62,76]. In colorectal adenocarcinoma, higher expression levels of PRKAA1, PRKAA2, PRKAB1, and PRKAB2 are associated with improved overall survival compared to lower expression levels [77], suggesting that the expression of the aforementioned genes is dependent on both the tumor type and the biological context. 

The final group of genes differentiating G3/G4 samples from G2 samples is *PER1-3* mRNAs, the expression of which was found to decrease with increasing tumor grade. Methylation pattern analysis showed that, irrespective of astrocytic tumor grade, the promoter regions of the *PER1-3* genes were methylated in all samples, which may explain the silencing of their expression. Additionally, expression of *PER1* and *PER2* is regulated by hsa-miR-24-3p, which we noted was overexpressed. This overexpression may explain why, with increasing astrocytic tumor stage, the levels of these proteins were lower compared to lesser stages. Moreover, other researchers have found lower PER1/2 expression in in higher-grade gliomas [78,79,80]. Furthermore, hsa-miR-24-3p has been reported to be overexpressed in brain tumors, which contributes to promoting tumor cell proliferation and enhancing angiogenesis [81,82].

Despite the comprehensive approach taken in this study, several limitations must be acknowledged.

First, the number of patients and samples was 60. This limited sample size may affect the generalizability of the findings. Additionally, this study focused exclusively on astrocytic tumors, which may not fully represent the diversity of tumors and their varying biological behaviors.

Second, while this study incorporated advanced molecular techniques such as microarray analysis and qRT-PCR, it did not include functional assays to validate the biological implications of the observed changes in gene expression. Functional validation through in vitro or in vivo models would provide more definitive evidence of the roles of circadian clock genes in astrocytic tumor progression.

Third, this study relied on methylation-specific PCR to analyze DNA methylation patterns. Although this method is widely used, it has limitations in sensitivity and may not detect all methylation changes, particularly those occurring at low frequencies. More advanced techniques, such as bisulfite sequencing, could offer a more detailed and comprehensive methylation profile.

Fourth, this study did not explore the potential impact of genetic variations or mutations in the circadian clock genes themselves, which could contribute to the pathogenesis of astrocytic tumors. Future studies could benefit from incorporating whole-genome sequencing or targeted sequencing approaches to identify such variations.

Moreover, the changes in the concentrations of selected proteins were determined using only one method: the ELISA assay. To enhance the reliability and comprehensiveness of our proteomic analysis, it is crucial to incorporate additional validation methods, such as Western blot analysis and immunohistochemistry. These techniques will provide complementary data and help confirm the observed changes in protein concentration, ensuring a more thorough and accurate understanding of the proteome dynamics.

In addition, while our data demonstrate significant associations of higher CLOCK gene and protein expression with higher tumor grades, and of the presence of methylated promoter regions of PER1-3 genes with lower expression levels, we agree that these correlations do not inherently imply causality. Although these associations are strong and suggestive, they do not establish a direct causal relationship. Potential confounding factors could have influenced the observed correlations, such as the tumor microenvironment, genetic heterogeneity among samples, and other molecular pathways that might interplay with circadian clock genes.

To address these limitations, we suggest future studies involving functional experiments. These could include knockdown or overexpression studies in cell lines and animal models to directly test the effects of circadian clock gene modulation on tumor behavior and progression.

Nevertheless, the method of preparing patients for the procedure, including fasting for at least 12 h beforehand and standardizing the time of the procedure, minimized the impact of indirect factors on the observed expression patterns of circadian clock genes and their encoded proteins, which depend on the time of day.

In summary, while this study provides valuable insights into the molecular underpinnings of astrocytic tumor progression, its limitations underscore the need for further research with larger samples, functional assays, more comprehensive methylation analyses, exploration of genetic variations, and clinical outcome correlations.

## 5. Conclusions

This study highlights the significant role of circadian clock genes in the progression of astrocytic tumors, with a particular focus on differences between low-grade (G2) and high-grade (G3/G4) tumors. We observed that the expression of the *CLOCK*, *PRKAA1*, *PRKAA2*, *PRKAB1*, *PRKAB2*, *PER1*, *PER2*, and *PER3* genes, as well as their corresponding proteins, varied significantly with the tumor grade. Notably, the *CLOCK* gene and its associated protein demonstrated increased expression in more advanced tumor stages, suggesting its potential role in tumor progression. Our findings indicate that DNA methylation patterns play a crucial role in the regulation of these circadian clock genes. All samples, regardless of their astrocytic tumor grade, exhibited methylation in the promoter regions of *PER1-3* genes, which likely contributed to the silencing of their expression. Additionally, this study identified hsa-miR-24-3p as a key regulator of *PER1* and *PER2* expression, with its overexpression correlating with decreased levels of these proteins in higher-grade tumors.

This study also underscores the complex regulatory mechanisms involving miRNAs such as hsa-miR-106-5p, hsa-miR-20b-5p, and hsa-miR-30d-3p, which impact the expression of circadian clock-related genes. These interactions highlight the intricate network of transcriptional and post-transcriptional regulation that influences astrocytic tumor progression.

While this study provides valuable insights into the molecular dynamics of circadian clock genes in astrocytic tumors, several limitations must also be acknowledged, including the relatively small sample size, the lack of functional validation, and the need for more advanced methylation analysis techniques. Future research should address these limitations and explore the clinical implications of these molecular changes in the diagnosis, prognosis, and treatment of astrocytic tumors. In conclusion, this research advances our understanding of the role of circadian clock genes in astrocytic tumor biology and underscores the potential of these genes as biomarkers for tumor progression and therapeutic targets. Further studies are warranted to validate and translate these findings into clinical applications that could improve patient outcomes in the management of astrocytic tumors.

## Figures and Tables

**Table 1:** Patient characteristics.

Gender	Age (Years)	WHO Grade of Malignancy	Number of Samples
Female (*n* = 32)	55.9 ± 3.4	G2	9
	57.9 ± 2.8	G3	5
	56.9 ± 2.2	G4	18
Male (*n* = 28)	58.8 ± 2.3	G2	7
	58.9 ± 1.7	G3	4
	56.4 ± 2.1	G4	17

G2—II grade; G3—III grade; G4—IV grade; WHO—World Health Organization; values of clinical parameters are presented as means ± standard deviation.

**Table 2:** qRT-PCR primers.

mRNA	qRT-PCR Amplification Primers (5′-3′)
*CLOCK*	Forward: AAAGTTAAGATTTTGGGTTAGATAAT
	Reverse: ACCATCTTCTCATAAACTAATAAATACTAC
*PRKAA1*	Forward: AGATTTAGTTTTTGGAGAAAGATGG
	Reverse: TTTATACCCAATCAATTCATATTTACC
*PRKAA2*	Forward: TTTGAAGATTTTTTTTATGATGTTAAC
	Reverse: ACTCACTAACTTAATTCATTATTCTCCG
*PRKAB1*	Forward: GAGTTTTTTGTTTAGGGTTTTTTTT
	Reverse: CCAAAAATTCCTCCTTCTCTAATAC
*PRKAB2*	Forward: TTATATTAGTGGTTTTTGGAGGAGG
	Reverse: CCCAAAAAACTTAAAATCAAAAAAAC
*PER1*	Forward: ATTTTGGAGGAGTTGGAGTATATTA
	Reverse: AAAAAACCAAAAACTCAAAAAAAC
*PER2*	Forward: GTGTGTTTTTGGTTTTGTTTTAGGT
	Reverse: AAACCACTACTCATATCCACATCTTC
*PER3*	Forward: GGTTGTAGGAAAGGGAAGTATAAG
	Reverse: AAAAAACCTAACTAAACACCATAAC
β-actin	Forward: TCACCCACACTGTGCCCATCTACGA
	Reverse: CAGCGGAACCGCTCATTGCCAATGG

*CLOCK*, clock circadian regulator; *PRKAA1*, protein kinase AMP-activated catalytic subunit alpha 1; *PRKAA2*, protein kinase AMP-activated catalytic subunit alpha 2; *PRKAB1*, protein kinase AMP-activated non-catalytic subunit beta 1; *PRKAB2*, protein kinase AMP-activated non-catalytic subunit beta 2; *PER1*, period circadian regulator 1; *PER2*, period circadian regulator 2; *PER3*, period circadian regulator 3.

**Table 3:** Characteristics of primers designed for the MSP.

mRNA		NCBI Reference Sequence	qRT-PCR Amplification Primers (5′-3′)
*CLOCK*	M	NM_001267843.2	Forward: TTAAGATTTTGGGTTAGATAATCGT Reverse: AAATAAAATACTCGTATCCGTCGAA
	U		Forward: TTAAGATTTTGGGTTAGATAATTGT Reverse: AAATAAAATACTCATATCCATCAAA
*PRKAA1*	M	NM_006251.6	Forward: GGTTGTTGAAATATTAAGGGTACGT Reverse: ACTTATCACAAAATTCTTCCTTCGTA
	U		Forward: GGTTGTTGAAATATTAAGGGTATGT Reverse: ACTTATCACAAAATTCTTCCTTCATA
*PRKAA2*	M	NM_006252.4	Forward: TTTGTTTGTTGTGGATTATTGTTATAG Reverse: TCCAAATATCAACTTCAAAACCTAC
	U		Forward: AGATGTTTATTGGATGTATTGAATA Reverse: CAAATAAAATTATAAACTCATTTTCAC
*PRKAB1*	M	NM_006253.5	Forward: GGTATGGTGGTTATAAGACGTTTC Reverse: TCTCTAATACCTTAATTTCCTCGAA
	U		Forward: GTATGGTGGTTATAAGATGTTTTGG Reverse: TCTCTAATACCTTAATTTCCTCAAA
*PRKAB2*	M	NM_005399.5	Forward: GTTTTGAAGGTAGGAGTGGAATTC Reverse: AAAACCTAAAATTCTCCAATACGAT
	U		Forward: TTTGAAGGTAGGAGTGGAATTTG Reverse: AAAACCTAAAATTCTCCAATACAAT
*PER1*	M	NM_002616.3	Forward: TGTCGTATTAGAGGAGGTTTTGATC Reverse: CAAAAAATATCCGAAAAACTTCGTA
	U		Forward: GTTGTATTAGAGGAGGTTTTGATTG Reverse: CAAAAAATATCCAAAAAACTTCATA
*PER2*	M	NM_022817.3	Forward: TTGAGTATATTGTGAAGAATGTCGA Reverse: TAACTTTTCCGAACACTAACACG
	U		Forward: TTGAGTATATTGTGAAGAATGTTGA Reverse: AACTTTTCCAAACACTAACACAAC
*PER3*	M	NM_001289862.2	Forward: GGTTGTAGGAAAGGGAAGTATAAGC Reverse: GACAAATAAAAAAATCGAACTCGAA
	U		Forward: TTGTAGGAAAGGGAAGTATAAGTGG Reverse: AACAAATAAAAAAATCAAACTCAAA

M, primers designed for methylated sequences; U, primers designed for non-methylated sequences. *CLOCK*, clock circadian regulator; *PRKAA1*, protein kinase AMP-activated catalytic subunit alpha 1; *PRKAA2*, protein kinase AMP-activated catalytic subunit alpha 2; *PRKAB1*, protein kinase AMP-activated non-catalytic subunit beta 1; *PRKAB2*, protein kinase AMP-activated non-catalytic subunit beta 2; *PER1*, period circadian regulator 1; *PER2*, period circadian regulator 2; *PER3*, pe-riod circadian regulator 3.

**Table 4:** The concentration of selected proteins related to the circadian clock k in the G2, G3, and G4 astrocytic tumor samples.

Protein	G2	G3	G4
CLOCK [ng/mL]	3.12 ± 0.19 ^A,B^	5.67 ± 0.65	9.87 ± 0.19 ^C^
PRKAA1 [ng/mL]	1.98 ± 0.27 ^A,B^	3.45 ± 0.18	6.67 ± 0.67 ^C^
PRKAA2 [ng/mL]	2.10 ± 0.19 ^B^	2.21 ± 0.65	4.45 ± 0.51
PRKAB1 [ng/mL]	9.81 ± 0.18	3.44 ± 0.34 ^A,B^	1.45 ± 0.12 ^C^
PRKAB2 [ng/mL]	412.01 ± 23.98 ^A,B^	654.11 ± 56.98	236.98 ± 43.81 ^C^
PER1 [ng/mL]	4.51 ± 0.18 ^A^	3.22 ± 0.19	2.91 ± 0.75 ^C^
PER2 [ng/mL]	4.76 ± 0.54 ^A,B^	4.56 ± 0.34	2.19 ± 0.23 ^C^
PER3 [ng/mL]	7.18 ± 0.98 ^A,B^	5.01 ± 0.18	2.88 ± 0.44 ^C^

CLOCK, clock circadian regulator; PRKAA1, protein kinase AMP-activated catalytic subunit alpha 1; PRKAA2, protein kinase AMP-activated catalytic subunit alpha 2; PRKAB1, protein kinase AMP-activated non-catalytic subunit beta 1; PRKAB2, protein kinase AMP-activated non-catalytic subunit beta 2; PER1, period circadian regulator 1; *PER2*, period circadian regulator 2; PER3, period circadian regulator 3; ^A^, statistically significance differences G2 vs. G3 (*p* < 0.05); ^B^, statistically significance differences G2 vs. G4 (*p* < 0.05); ^C^, statistically significance differences G2 vs. G3 (*p* < 0.05); G2—II grade; G3—III grade; G4—IV grade. Data are presented as the mean ± standard deviation.

## Data Availability

The data used to support this study’s findings are included in this article. The data will not be shared due to third-party rights and commercial confidentiality.
